# High Rate of Antibody Response to Multiple Doses of the COVID-19 Vaccine in Liver Transplant Recipients: Analysis of Predictive Factors

**DOI:** 10.3390/vaccines13040352

**Published:** 2025-03-26

**Authors:** Nunzio Zignani, Andrea Costantino, Michele Sagasta, Clara Dibenedetto, Riccardo Perbellini, Sara Uceda Renteria, Pietro Lampertico, Maria Francesca Donato

**Affiliations:** 1 Department of Pathophysiology and Transplantation, University of Milan, 20122 Milan, Italy; andrea.costantino@policlinico.mi.it (A.C.); pietro.lampertico@unimi.it (P.L.); 2 Gastroenterology and Endoscopy Unit, Foundation IRCCS Ca’ Granda Ospedale Maggiore Policlinico, 20122 Milan, Italy; 3 Gastroenterology and Hepatology Unit, Foundation IRCCS Ca’ Granda Ospedale Maggiore Policlinico, 20122 Milan, Italy; michele.sagasta@policlinico.mi.it (M.S.); clara.dibenedetto@policlinico.mi.it (C.D.); riccardo.perbellini@policlinico.mi.it (R.P.); francesca.donato@policlinico.mi.it (M.F.D.); 4 Microbiology and Virology Unit, Foundation IRCCS Ca’ Granda Ospedale Maggiore Policlinico, 20122 Milan, Italy; sara.ucedarenteria@policlinico.mi.it

**Keywords:** liver transplant recipients, COVID-19, vaccine hesitancy, vaccination, first booster, second booster, fourth dose, predictive factors

## Abstract

Background: The COVID-19 pandemic highlighted the vulnerability of immunocompromised individuals, including liver transplant recipients (LTRs), who often exhibit reduced vaccine immunogenicity. While initial vaccine doses and subsequent boosters improved immune response, LTRs were prioritized for vaccination. Studies have shown increased antibody response after each booster dose. Vaccine hesitancy, defined as delayed or refused vaccination despite availability, poses a public health challenge, often fueled by misinformation. This study aimed to evaluate anti-spike antibody responses in vaccinated LTRs after two initial doses and at least one booster, also assessing adherence to subsequent doses. Methods: We conducted a retrospective observational study at a transplant center in Milan, Italy, between January 2021 and December 2023. LTRs who had received four or more doses of mRNA vaccines (Pfizer or Moderna) were included. Anti-spike antibody levels were measured 60–80 days after each dose. Data on vaccination status were collected in January 2024. Statistical analysis was performed to compare antibody responses and identify predictive factors. Results: LTRs showed a significant increase in anti-spike antibody responses after the first booster compared to the second dose with a trend versus a further increase following the fourth dose in a subgroup of the patients receiving two booster doses. However, adherence to booster doses decreased over time. In LTRs, predictors of a weaker response after the second dose were chronic kidney disease and metabolic etiology at transplant. Conclusions: The study highlighted that in LTRs, multiple doses of the COVID-19 vaccine led to a continuous increase in anti-spike antibody responses. The progressive decline in adherence of LTRs “to further booster doses” should be related to the fact that after the spread of vaccination programs worldwide, COVID-19 is still a current infection, but it is much less severe than before.

## 1. Introduction

### 1.1. COVID-19 Vaccination

The coronavirus disease 2019 (COVID-19) pandemic caused by severe acute respiratory syndrome coronavirus 2 (SARS-CoV-2) was the worst pandemic of this century. The disease is characterized by fever, cough, myalgia, and respiratory issues of varying severity, ranging from mild dyspnea to potentially fatal acute respiratory failure. Nevertheless, a significant proportion of patients may experience an asymptomatic course of the disease [1]. To counteract the spread and severity of the virus, research for vaccine development began in 2020, and within less than a year, many vaccines were approved by the WHO through emergency procedures [2]. The developed vaccines employ different mechanisms of action: attenuated or inactivated virus, protein subunit, viral vector, DNA or mRNA, or virus-like particles (VLP). Moreover, it has been demonstrated that mRNA vaccines exhibit the highest reactogenicity, followed by viral vector and protein subunit vaccines [3]. Despite the fact that most commercially available vaccines have yielded excellent results in terms of efficacy [4], data indicate that the COVID-19 vaccine could be less immunogenic in immunosuppressed transplant recipients [5,6,7,8]. Administration of the first two doses of the SARS-CoV-2 vaccine began at the end of 2020, and the subsequent booster doses proved effective in improving the immune response to the virus [9]. Liver transplant recipients (LTRs) and solid organ recipients, as immunocompromised by anti-reject therapy, were among the priority candidates for the vaccine (from the end of 2020 until March 2022 with the fourth dose) [10,11]. The indication for vaccination in liver transplant patients was unequivocally shared by the Ministry of Health and other public health authorities [12].

### 1.2. Immunization in Liver Transplant Recipients

It has been demonstrated in numerous studies that the immune response is increased after the first booster dose compared to the first two doses and also after the second booster dose compared to the previous doses, both in the humoral response (considering anti-SARS-CoV-2 antibodies) and in the cell-mediated response (considering T lymphocytes) [13,14,15,16,17,18,19,20]. Furthermore, it has been demonstrated that the immune response to the vaccine is better in LTRs than in other transplant recipients, such as kidney [21] or lung [19,22] transplant recipients, due to different immunosuppressive regimens and comorbidities. On the other hand, the decrease in the coverage given by the immune system against the virus concerns both healthy patients and patients who are immunosuppressed due to diseases or immunosuppressive therapies, as in the case of patients undergoing solid organ transplants [23,24].

### 1.3. Vaccination Hesitancy

According to the Strategic Advisory Group of Experts on Immunization (SAGE) of the World Health Organization (WHO), the term “vaccination hesitancy” refers to “delay in accepting or refusing vaccination despite availability” [25]. Non-adherence to the vaccine in the population represents a health problem because the decision not to get vaccinated is often given by uncertainty about adverse events, in turn, caused by incorrect information or fake news collected by patients from inadequate sources [26,27,28,29], while in some patients, the motivation may stem from fear of injection [30]. Vaccine hesitancy also represents a threat to global health, with coverage declining, putting at risk the advantage of herd immunity, which is positive for patients who cannot get vaccinated or who are unable to mount an adequate protective immune response. Adherence varies in the population based on various factors, such as geographical or socio-cultural ones. A review by Yasmin et al. [31] identified in the United States a lower adherence in the black ethnic group, in patients <45 years, in pregnant women, and in the population of the lowest income groups, while a higher adherence in whites and Asians, in the population >45 years and with higher income. Despite this evidence, adherence, in general, stood at approximately 78% and has been shown to increase over time during the vaccination campaign [32,33].

### 1.4. Aims of the Study

The aims of our study were to evaluate in our cohort of vaccinated LTRs: 1. the humoral response rates of anti-SARS-CoV-2 spike protein after the two standard doses and two additional boosters 2, the predictors of antibody response, and 3. the vaccine adherence to doses after the two additional booster doses.

## 2. Materials and Methods

### 2.1. Study Design and Participants

A retrospective observational study was conducted on LTRs, followed at the Transplant Clinic of the Gastroenterology and Hepatology Division, Foundation IRCCS Ca’ Granda Ospedale Policlinico of Milan, between January 2021 and December 2023. All patients received at least three doses of mRNA vaccines (Pfizer/Comirnaty BNT162b2, Pfizer, New York, NY, USA; Moderna/Spikevax mRNA-1273, Moderna, Cambridge, MA, USA).

### 2.2. Determination of Anti-SARS-CoV-2 Antibodies

Humoral immune response was measured 60–80 days after each dose of vaccine by using electrochemiluminescence immunoassay “ECLIA” for the quantitative detection of antibodies to SARS-CoV-2 spike protein RBD (targeting the receptor binding domain, RBD) at each timepoint. A SARS-CoV-2 infection during the study period was diagnosticated with positivity of nasopharyngeal swab to SARS-CoV-2 RNA with SARS-CoV-2 PCR assay or antigenic test and/or according to the presence of anti-SARS-CoV-2-Nucleocapsid IgG (anti-N).

#### 2.2.1. Serological Assay

The quantification of anti-SARS-CoV-2 antibody titers was analyzed with two commercially available electrochemiluminescence immunoassay (ECLIA), Elecsys Anti-SARS-CoV-2 S and Elecsys Anti-SARS-CoV-2 (Roche Diagnostic, Basel, Switzerland) on the cobas e 801 immunoassay analyzers. Serum samples were processed and analyzed according to the manufacturer’s protocol. Elecsys Anti-SARS-CoV-2 S was used to evaluate the SARS-CoV-2 humoral immune response elicited by BNT16262 vaccine by the quantitative detection of antibodies to SARS-CoV-2 spike protein RBD (anti-RBD antibodies) total Ig at each timepoint: baseline, 60 days after the second, third, and fourth dose in our cohort of LTRs. The Elecsys Anti-SARS-CoV-2 S assay uses a recombinant protein representing the RBD of the spike antigen, detecting anti-SARS-CoV-2 spike protein RBD antibodies.

The analytical measuring interval is 0.40–250 U/mL. The lower limit of quantification (LLOQ) was 0.4 U/mL; the upper limit of quantification (ULOQ) was 12,500 U/mL for 50-fold diluted samples. Numeric values <0.8 U/mL were interpreted as negative, and numeric values ≥0.8 U/mL were interpreted as positive per anti-SARS-CoV-2 spike protein RBD antibodies.

In addition, another test, Elecsys Anti-SARS-CoV-2 assay, was used for the qualitative determination of antibodies against SARS-CoV-2 nucleocapsid protein (anti-N) total Ig at baseline and during the overall period of follow-up to exclude asymptomatic infection in LTRs or to demonstrate a prior SARS infection.

The Elecsys Anti-SARS-CoV-2 assay utilizes a recombinant nucleocapsid (N) antigen protein to facilitate the detection of anti-SARS-CoV-2 nucleocapside (anti-N) antibodies with high affinity. A cutoff index (COI) ≥ 1.0 was interpreted as positive for anti-SARS-CoV-2 nucleocapside (anti-N) antibodies.

#### 2.2.2. rRT-PCR SARS-CoV-2 RNA Assay and Antigenic Test

Nasopharyngeal swabs from suspected SARS-CoV-2 infected LTRs underwent Real-time reverse transcription polymerase chain reaction (rRT-PCR) testing for SARS-CoV-2 RNA with Allplex SARS-CoV-2 assay on CFX96 platform (Seegene, Seoul, Republic of Korea).

Furthermore, antigenic test, STANDARD F COVID-19 Ag FIA, was used to diagnose SARS-CoV-2 infection in patients with clinical symptoms of SARS-CoV-2 infection. STANDARD F COVID-19 Ag is the fluorescent immunoassay for the qualitative detection of specific nucleoprotein antigens to SARS-CoV-2 present in nasopharyngeal swabs and was used with the STANDARD F Analyzers (SD BIOSENSOR, Suwon-si, Republic of Korea).

### 2.3. Statistical Analysis

The following clinical and epidemiological variables were recorded: baseline features (sex, age, and age at transplant), liver disease etiology (viral, autoimmune-sclerosing cholangitis-primary biliary cholangitis (AIH-PSC-PBC), MASLD (metabolic dysfunction-associated steatotic liver disease, indications for transplantation (hepatocellular carcinoma, decompensated cirrhosis, fulminant hepatitis), immunosuppressive therapy (Tacrolimus monotherapy, Tacrolimus and Micofenolate/Azathioprine or steroid or Sirolimus/Everolimus, or triple therapy) and comorbidities. Data were analyzed with R statistical programming software (R Core Team (4.2.1, Vienna, Austria)) using the package R Commander (“rcmdr”).

Categorical variables are shown with frequency and percentage, while continuous variables are shown with median and interquartile range. Quantitative antibody responses are shown with mean and standard deviation (SD), while the difference between responses at various doses was calculated with McNemar’s test with continuity correction for categorical assessment (percentage of responders) and with paired *t*-test for quantitative assessment (antibody titers) setting a significance level of α = 0.05.

Univariate predictive analysis was conducted using logistic regression, with the qualitative vaccine response after the second dose as the explanatory variable (responder or not responder) and the other variables as predictive, to assess which were most predictive of antibody response.

Before every analysis, any antibody values that fell below the LLOQ were replaced with a value equal to 0.5 times the LLOQ. If any values exceeded the ULOQ and the actual values were not available, they were substituted with the ULOQ. Liver graft function of our transplanted patients at the time of first vaccination was evaluated by Fibroscan (Echosens, Paris, France), considering a normal value <6.9 kPa. Data on vaccination status were collected in January 2024; the reasons for non-adherence were collected in 70% of all patients.

## 3. Results

### 3.1. Immunization

We enrolled 162 out of 245 liver transplant recipients (66%) who had been vaccinated for COVID-19 and followed in our Unit, of whom all serological data were available. As shown in Table 1, most patients were males of advanced age at the time of vaccination and were transplanted for viral liver diseases. Immunosuppression was CNI-based in all patients. Tacrolimus was given in monotherapy or combined with other immunosuppressive drugs. Most patients exhibited a good value of liver stiffness by fibroscan (median 6 Kp, IQ range 4.5–7.8). Among the comorbidities, hypertension represented the most common disease by far.

**Figure 1:**
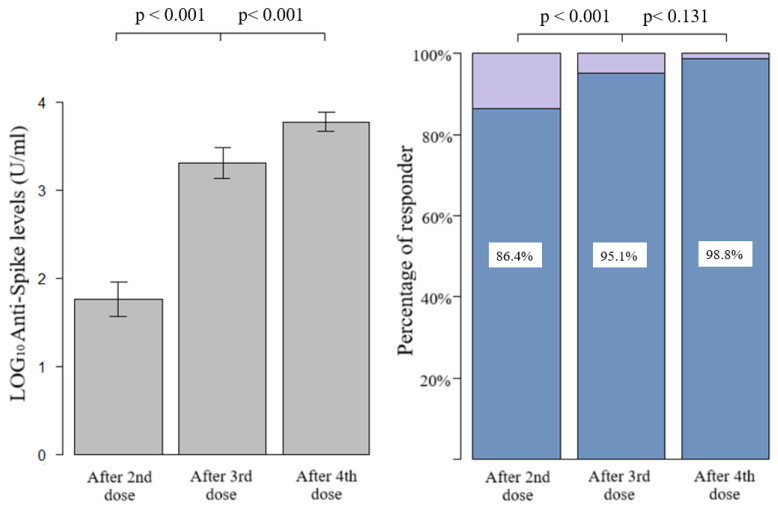
Bar graph of quantitative and qualitative anti-spike response after second, third, and fourth dose, compared with paired *t*-test (quantitative) and McNemar test with continuity correction (qualitative).

**Figure 2:**
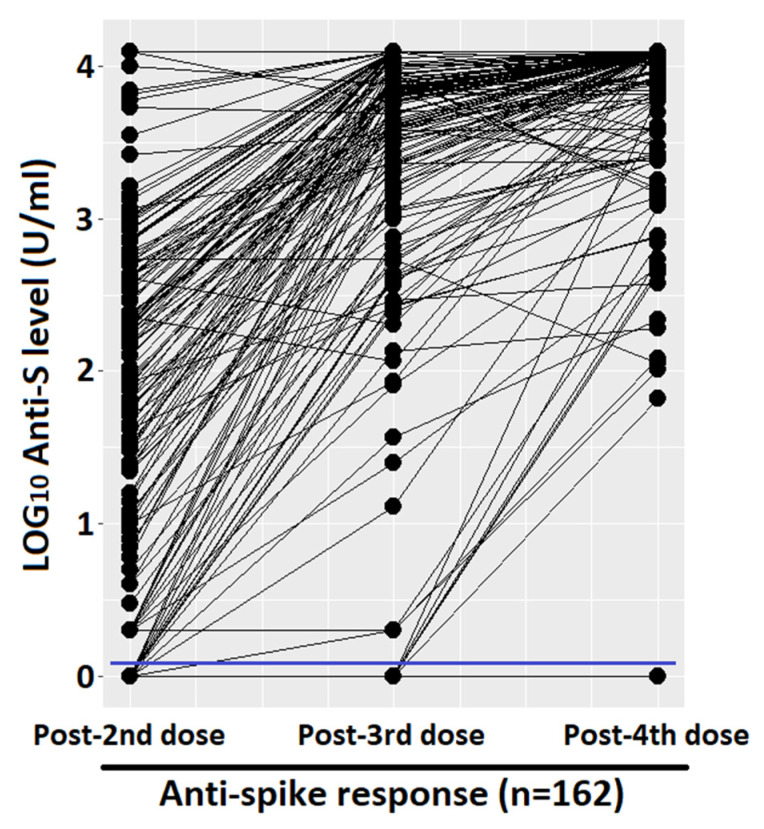
Paired dot plot that compares quantitative anti-spike response, measured with LOG_10_ level. Cut-off of 0.8 (U/mL) was marked with a blue line.

Figure 1 shows the quantitative humoral response and the percentage of responder. After the first two doses, we had an average of 1.76 LOG_10_ levels of anti-spike (SD 1.27), with a progressive and significant increase after the third and fourth doses (average of 3.30 LOG_10_, SD 1.14, and 3.77 LOG_10_, SD 0.6, respectively). The prevalence of qualitative response (antibody response cut-off = 0.8 U/mL) in LTRs after the third dose was significantly higher with respect to those vaccinated with two doses (95.1%, CI 91.8–98.4%, vs. 86.4%, CI 81.1–91.7%, *p*-value < 0.001, respectively). Due to the high antibody responses achieved after both the third and fourth doses (95.1% and 98.4%), the difference between the responses was not statistically significant at a level α = 0.05, despite a clear increase in quantitative antibody responses in almost all the samples, as seen in the paired dot plot shown in Figure 2. Considering that the concomitant COVID-19 infection during the study period could represent a confounding factor, the LTRs who tested positive at the nasopharyngeal test for SARS-CoV-2 were stratified according to the number of doses received: (1) one infection before the first two vaccinations, (2) no infection after the first two doses, (3) 17 infections following the third dose, none of which were considered serious according to criteria reported in the methodological section, (4) 34 infections after the fourth dose including one serious and one death. Among the patients who did not respond to the vaccine, only one had the infection (not serious, which occurred after the third dose).

A univariate predictive analysis was conducted to assess potential factors that could predict the antibody response to the vaccine. Table 2 shows the univariate analysis after the second vaccine dose where chronic kidney disease and metabolic etiology at transplant resulted predictive of a weaker antibody response to the second vaccine dose. We cannot use the presence of coinfection with SARS-CoV-2 as a predictive variable because there was only one infection in the period before the second dose, the period covered by the predictive analysis. The limited number of non-responder patients in subsequent doses (eight after the third dose and two after the fourth dose, respectively) hindered a statistical evaluation and confirmed the efficacy of at least three doses of vaccine. Among the analyzed variables, it is worth noting that it was not possible to construct a contingency table for patients transplanted due to fulminant hepatitis due to the small number of cases.

### 3.2. COVID-19 Vaccine Hesitancy

We observed an optimal adherence of our cohort of LTRs to vaccine boosters up to the fourth dose (95.6%). Thereafter, the rates of vaccination dropped to 77.5% for the fifth and to 20.8% for the sixth booster doses.

## 4. Discussion

Our data on liver transplant setting confirm the importance of consecutive vaccinations for COVID-19, which, as expected, recalls the normal clinical practice of many other infectious diseases: a renewal of the immune stimulus given by the vaccine is necessary to maintain adequate levels of response and avoid severe forms of a potentially fatal disease. In fact, our study demonstrates a continuous increase in the antibody response from the second to the third and fourth doses. These data are in line with the literature [13,14,15,16,18], although some differences in the response can be highlighted: the prevalence of responder patients is higher after the second dose and at the upper limits following the third, compared to the majority of studies which attest a response between 66% and 71% following the first vaccination cycle [13,14,15,20,34] and between 84% and 98% [13,14,15,17,18] after the first booster. Possible reasons for these discrepant results could be the different studied populations and the sample size of the studies. Another reason could be the time lag between vaccine dose and serological test, a figure not always present in the literature and characterized by a certain variability between studies and within the same study. Indeed, some studies in the literature suggest that immunocompromised patients take more time to mount an adequate antibody response following vaccination. This is why response levels could be directly proportional to the time elapsed between administration and serological testing [35,36].

Furthermore, the high prevalence of responders after the third dose, as mentioned in the results, is the cause of a lack of significance in the difference between the third and fourth doses, a clinically clear difference but too low to satisfy purely statistical criteria. The response of our LTRs to the fourth dose of SARS-CoV-2 vaccine is in line with the published data [17,18], and the range presented by the various studies is narrower and tends to be 100%.

Regarding predictive variables, the small number of non-responder cases led to limited outcomes. Chronic kidney insufficiency, in agreement with the literature [35,37], was predictive of a lower antibody response to the second dose of the vaccine due to its dysregulating effect on the immune system and a possible effect of therapies such as mycophenolate mofetil or steroids [38]. Age did not exhibit any relationship with the antibody response, although some studies showed that advanced age predicts a weaker immune response [6,37]. Similarly, no relationship was found between liver transplant timing and antibody response, a link present in some studies [37,39] but absent in others [35]. Triple immunosuppressive therapy or dual therapy showed a negative β value in the absence of statistical significance. This could be attributed to limited statistical power, given that data reported in the literature emphasize that higher immunosuppression and, in particular, the use of combo therapy with Mycophenolate mofetil or steroids leads to a reduced antibody response [35,37,38,39,40,41]. It should be emphasized that our data showed a reduced antibody response towards the second vaccine dose in liver transplant recipients with metabolic etiology, including other components of metabolic syndrome, such as diabetes mellitus and dyslipidemia, overweight, and alcohol abuse. This relationship could be explained by the synergy between the underlying disease and immunosuppressive therapy, which could influence the antibody response. In fact, the role of diabetes and obesity in suppressing the immune system by promoting a pro-inflammatory state and affecting leukocyte activity is already known [42,43]. Unfortunately, the literature lacks data on the relationship between metabolic etiology and vaccine non-response. The lack of predictive variables for antibody response from the third and fourth boosters of the vaccine can be explained by the high prevalence of antibody responses achieved.

In conclusion, it can also be considered that the antibody responses to the SARS-CoV-2 vaccine in liver transplanted patients are high in relation to the status of “immunocompromised patient” because it is known that these patients are subject to lighter immunosuppressive therapies compared to other solid organ transplant recipients [44]. This leads to better general immune responses, in terms of humoral and cellular [5,14] immunoreactivity to vaccines and reduces the high risk of disease severity [10,11,45], although this effect is not comparable to the setting of immunocompetent patients [18,23,24].

### 4.1. Limitations of the Study

There are some limitations in this study to be aware of, such as the following: (1) the interval between the administration of the vaccine and the blood draw for the serological test is variable, and also the ranges present in the literature; this could influence the antibody response, which, as we know, is time dependent. (2) The lack of quantitative determination of the patients’ antibodies above the limit threshold of 12,500 U/mL did not allow us to calculate the averages and medians correctly, and consequently, quantitative comparison of antibody responses following doses using the paired *t*-test may be inaccurate, which is why the percentage of responding patients has been shown in Figure 1, on which inferences were made using the McNemar test. (3) A predictive analysis using linear regression with quantitative antibody response (actual anti-spike levels following each dose) was not conducted. This is because, unlike logistic regression which assessed the distinction between responder and non-responder patients, the antibody levels within the positive class span an interval of four powers of ten (from 0.8 to 12,500, approximately 10^1^ to 10^4^). Consequently, in the calculation of the beta estimate, negative patients lose statistical significance entirely. To simplify, a patient with the maximum response (ULOQ) weighs significantly more in the model than a patient with a medium/low response or a non-responder. This introduces a bias, as the model’s output is predominantly influenced by the high values of a subset of patients within the positive class.

### 4.2. Vaccine Hesitancy

Adherence to doses following the first three showed a clear decrease in the general population and also in the immunocompromised population, but this should not be confused with hesitancy. In fact, the main cause is represented by intercurrent infections (remember that the infectious waves have progressively grown in the absolute number of infected), which have decreased the possibility of carrying out subsequent doses of the vaccine, according to the indications of the Ministry of Health [46] for the postponement of the recall following the infection. This mechanism, according to some patients, led to avoiding the execution of the booster by postponing it to the following autumn due to the lowering of the infection curve and the consequent lack of sense of necessity given by the period of low virulence (such as the summer period). A secondary cause, despite its importance, is the actual distrust of patients towards the vaccine in general. This is a situation with different nuances, not as clear as the clear refusal of patients who have never received a vaccine dose [47], and must be addressed from certain points of view: in fact, some patients say they had many doubts about the indication from the beginning, despite the awareness of being a class at risk of infection, but having nevertheless carried out the first doses and the first booster. For the subsequent doses, the situation was different: first of all, the sense of necessity disappeared with the easing of the severity of the virus [48], and this could have been added to the aforementioned fear of needles [3], leading to a decision to give up due to the personal pro/con balance, and secondly, many patients arrived at the moment of the fourth or fifth dose when the obligation of green pass to access public facilities for work or other reasons had been revoked. These factors led already undecided patients to avoid doses following the third or fourth. For these reasons, it is not possible to consider vaccine hesitancy by simply evaluating what percentage of patients have reached the fourth or subsequent doses, but everything must be contextualized with other factors, sometimes more important, such as infections occurring between one dose and the next.

## 5. Conclusions

The data presented in this study show a higher prevalence of antibody response to SARS-CoV-2 vaccine doses among liver transplant recipients compared to previous studies. Metabolic etiology can be considered as a new predictor of response, although these data require further confirmation.

The usefulness and cost-effectiveness of these vaccines have already been widely demonstrated in the literature, both on the general population and on the population of transplanted patients [10,11,49], but follow-up data for doses subsequent to the fourth can corroborate the already strong indication of COVID-19 vaccine periodic recalls in solid organ recipients.

## Figures and Tables

**Table 1:** Baseline characteristics with etiology leading to liver transplantation and its indication, comorbidities (mainly cardiovascular risk), and immunosuppressive therapy.

Baseline Features	Patients = 162
Males (n/%)	113 (69.7%)
Age at vaccination (median, IQ range)	67 (59–71)
Age at transplant (median, IQ range)	57 (49–62)
Fibroscan (median, IQ range)	6 (4.5–7.8)
**Liver disease etiology (n, %)**	
Viral	100 (61.7%)
AIH-PSC-PBC	21 (13.0%)
MASLD	32 (19.8%)
Other	9 (5.5%)
**Indications for transplantation (n, %)**	
Hepatocellular carcinoma	80 (49.4%)
Decompensated cirrhosis	77 (47.5%)
Fulminant hepatitis	5 (3.1%)
**Comorbidities (n, %)**	
Arterial hypertension	122 (75.3%)
Diabetes	55 (34.0%)
Lung diseases	38 (23.5%)
Neoplasm	37 (22.8%)
Chronic kidney disease	28 (17.3%)
Obesity	28 (17.3%)
Cardiovascular diseases	23 (14.2%)
**Immunosuppressive therapy (n, %)**	
Tacrolimus (Monotherapy)	24 (14.8%)
Double therapy	127 (78.4%)
Triple therapy	11 (6.8%)

*Note*: AIH: autoimmune hepatitis; PSC: primary sclerosing cholangitis; PBC: primary biliary cholangitis; MASLD: metabolic dysfunction-associated steatotic liver disease.

**Table 2:** Univariate predictive analysis performed with logistic regression using qualitative anti-spike response as outcome variable.

Univariate Predictive Analysis					
Baseline Characteristics	β Estimate	OR	95% CI	OR	*p*-Value
Males	0.085	1.090	0.414	2.860	0.863
Age at vaccination	0.015	1.020	0.972	1.060	0.501
Age at transplantation	−0.019	0.981	0.935	1.030	0.442
Liver stiffness (Fibroscan)	0.107	1.11	0.945	1.310	0.199
**Comorbidities**					
Cardiovascular disease	0.568	1.760	0.384	8.110	0.466
Diabetes	−0.347	0.707	0.282	1.770	0.460
Hepatocellular carcinoma	−0.240	0.787	0.319	1.940	0.603
Arterial hypertension	−0.822	0.440	0.123	1.570	0.206
Chronic kidney disease	−1.731	0.177	0.0668	0.469	<0.001
Neoplasm	−0.022	0.978	0.305	3.140	0.971
Obesity	−0.699	0.497	0.175	1.410	0.189
Lung disease	0.048	1.050	0.359	3.060	0.931
**Etiology**					
Viral	0.770	2.160	0.872	5.350	0.096
MASLD	−1.016	0.362	0.137	0.958	0.004
AIH-PSC-PBC	−0.068	0.934	0.251	3.480	0.919
Other	0.241	1.270	0.151	10.700	0.824
**Transplant indications**					
Decompensated cirrhosis	0.097	1.100	0.447	2.720	0.834
Hepatocellular carcinoma	−0.240	0.787	0.319	1.940	0.603
Fulminant hepatitis					
**Immunosuppressive therapy**					
Tacrolimus	1.418	4.130	0.529	32.200	0.176
Double therapy	−0.247	0.781	0.246	2.480	0.675
Triple therapy	−0.958	0.384	0.094	1.570	0.184

*Note*: AIH: autoimmune hepatitis; PSC: primary sclerosing cholangitis; PBC: primary biliary cholangitis; MASLD: metabolic dysfunction-associated steatotic liver disease.

## Data Availability

The datasets generated and/or analyzed during the current study are not publicly available but are available from the corresponding author upon reasonable request.

## Correction Statement

Following the removal of this article from the Special Issue 3rd Edition: Safety and Autoimmune Response to SARS-CoV-2 Vaccination, an Editorial Board member reviewed the entire editorial process and confirmed all elements had been carried out as per MDPI Editorial Process policy (https://www.mdpi.com/editorial_process). As a result, the original Academic Editor listed on this publication has been replaced with that of the Editorial Board member who conducted this review. The original publication has now been updated accordingly. This information has also been transmitted to relevant indexing organizations.
